# Patient Pathways During Acute in-Hospital Stroke Treatment: A Qualitative Multi-Method Study

**DOI:** 10.5334/ijic.5657

**Published:** 2022-02-21

**Authors:** Loraine Busetto, Johanna Hoffmann, Christina Stang, Hemasse Amiri, Fatih Seker, Jan Purrucker, Peter Arthur Ringleb, Simon Nagel, Martin Bendszus, Wolfgang Wick, Christoph Gumbinger

**Affiliations:** 1 Department of Neurology, Heidelberg University Hospital, Im Neuenheimer Feld 400, 69120 Heidelberg, Germany; 2 Department of Neuroradiology, Heidelberg University Hospital, Im Neuenheimer Feld 400, 69120 Heidelberg, Germany; 3 Clinical Cooperation Unit Neuro-Oncology, German Cancer Research Center, Heidelberg, Germany

**Keywords:** patient pathways, stroke, endovascular treatment, process description, qualitative research

## Abstract

**Introduction:**

Patients experiencing acute ischemic stroke should access treatment as soon as possible to increase their chances for survival without severe disability. Given the increased complexity of stroke treatment from the provider and patient perspective, this study provides an overview of the pathways followed by stroke patients during in-hospital treatment.

**Methods:**

This qualitative study combined twenty-seven observations and fifteen staff interviews at a German comprehensive stroke center providing endovascular treatment (“EVT hospital”). Analysis was based on the COMIC Model for the comprehensive evaluation of complex health care interventions and a grounded theory approach.

**Results:**

The patient pathways during in-hospital treatment span the phases (1) admission to hospital, (2) receiving recanalization therapies, and (3) in-patient treatment. Before admission to the EVT hospital, interactions between staff members from the EVT hospital and patients take place as part of the telestroke consultations during which the EVT hospital’s ED neurologist meets the patient via a video- and audio-based connection. During the second phase, when IVT and/or EVT are provided to the patient, three teams (ED, neuroradiology and ICU team) with direct patient interactions intersect at the angiography suite until mechanical recanalisation treatment ends and the patient is transferred to the SU or ICU. In the third phase, the patients are treated on the SU or ICU and staff members interact with them according to a pre-defined schedule as well as based on individual needs.

**Discussion:**

Our results show that most direct staff-patient interactions are focussed within one phase, with a smaller number of interactions extending to other phases, and no professional (group) with direct patient interactions cover more than two phases of the acute stroke pathway. Future research should investigate how the pathways described here are experienced from the patient perspective, including how the organisation of visible care processes may influence patient satisfaction. Findings can be translated to accessible patient information resources as well as input for digitalisation efforts, provider orientation and training.

## Introduction

Acute ischemic stroke is one of the leading causes of death and disability worldwide, with the disease burden anticipated to increase even further over the next decades [1,2,3,4]. If a person experiences a stroke, they are likely to transition through almost all sectors of a given health system: starting with emergency services at symptom onset, hospital-based emergency treatment, in-patient treatment at a stroke unit and/or intensive care unit, discharge to a rehabilitation facility, community-based or primary care, and possibly permanent transition to a long-term care facility [5,6,7,8,9,10,11]. Patients will also be recommended to receive preventative care and to engage in self-management as they are now at increased risk for a second stroke [5,6,7,12,13,14].

Integrated care interventions for stroke have primarily focussed on problems in the post-acute phases of care such as transitioning from the hospital to care provision in the community or primary care setting. Examples described in the literature include “silo-based” care provision, lack of integration between sectors, limited access to specialists, gaps in care transitions and discharge planning as well as insufficient patient involvement, shared decision-making and self-management support [15,16,17,18,19]. However, even *within* hospitals, stroke care is extremely complex and fragmentation regularly occurs at various points along the pathway. As soon as the patient arrives, a team of health professionals from different specialties, professional groups and wards will start treating the patient so as not to lose valuable time, and thereby, brain function [20,21,22]. Since 2015, this process has become even more complex when endovascular thrombectomy became an additional evidenced based treatment option [23]. As explained in more detail below, this treatment option can currently only be provided at specialised hospitals and often requires additional (brain) imaging procedures and for eligible patients (outside specialised hospitals), emergency inter-hospital transfers from less to more specialised hospitals (see ***Information Box 1***).

**Information box 1 Acute stroke treatment**

Patients experiencing acute ischemic stroke can be treated using intravenous thrombolysis (IVT), which aims to dissolve the blood clot medicinally, and/or endovascular thrombectomy (EVT), which uses a wire mesh or an aspiration catheter for the mechanical removal of the blood clot [30,31,32,33,34]. Both IVT and EVT have time-dependent treatment effects and must therefore be administered as soon as possible after symptom onset [35,36,37]. IVT has been an established treatment option for almost 20 years and can be administered safely at all hospitals with a Stroke Unit (SU). Given the importance of fast treatment access, many countries already operate health systems in which SUs are distributed geographically, so that hospitals with a Stroke Unit can be accessed on time even from remote areas. In contrast, EVT has only been an evidence-based treatment option since 2015 and the organisation of whether and how EVT can be provided to all stroke patients who need it is still in an implementation phase. Currently, EVT can usually only be provided at a smaller number of geographically more dispersed specialist hospitals with the necessary personnel and infrastructural resources. Specifically, even though Germany has one of the highest SU-bed-to-person ratios in the world, 150/340 certified SUs are not able to perform EVT on a regular basis [38,39].

To ensure that all patients have access to optimal care – regardless of the geographic location of stroke onset – a large-scale re-organisation of acute stroke care is under way. This is primarily the case in higher income countries, but with reorganisation of emergency systems and more cooperation between hospitals, now increasingly also globally [40,41]. Most notably, hospitals have begun to organise in regional stroke networks with standardised procedures for cooperation around hyper-acute patient transfers for EVT [42,43,44]. For EVT-capable hospitals (“EVT hospitals”) this means an increased intake of patients not only from emergency services but also as emergency transfers from smaller hospitals performing IVT only (“IVT hospitals”). Moreover, once patients are admitted to an EVT hospital, a complex process to evaluate if a patient might benefit from EVT, differential diagnoses and contingent treatment pathways is started. Evaluation of eligibility and initiation of EVT has to be conducted under extremely high time pressure. As this requires the coordination and integration of different providers, departments, and professional groups, (acute) stroke treatment can be seen as an example of care integration [45].

Given this degree of complexity, operating well-integrated pathways is important for achieving optimal health outcomes as well as good patient experiences, adequate staff satisfaction and a (cost) effective organisation of care at the hospital level [24,25,26,27,28]. However, despite the important role of care integration for stroke care, its acute phase is usually primarily framed as a medical intervention. Detailed process descriptions are available in the form of standard operating procedures, providing detailed step-by-step instructions on who should perform which task when, where and how, but usually also with an almost exclusive medical focus [29]. Moreover, their intended target groups are hospital staff, not patients and relatives.

This manuscript works from the assumption that acute stroke care is a form of integrated care provision, and that framing and describing it as such will provide important insights for how it can be evaluated and improved. To this purpose, this manuscript aims to answer the following research question: What are the patient pathways in acute stroke, including interactions with different health professional groups, from the perspective of stroke patients? In doing so, we aim to centre the perspective of the patient (and where appropriate, their relatives) alongside the medical and professional perspective. Moreover, we aim to report our findings in a way that is useable for patient information and staff education purposes as well as for organisational-level improvement efforts.

## Methods

This qualitative single-centre study used a consecutive qualitative multi-method design, including non-participant observations and semi-structured interviews, based on the COMIC Model for the comprehensive evaluation of complex health care interventions and using a grounded theory approach [46,47]. We followed the Standards for Reporting Qualitative Research (SRQR) guidelines [48]. Ethics approval was obtained from the ethics commission of the Medical Faculty of Heidelberg University (S-682/2017). All interviewees provided informed consent before participating in the study.

### Setting

Data was collected at a university hospital in the south-west of Germany (Department of Neurology, Heidelberg University Hospital), which is a high-volume stroke centre providing both IVT and EVT. It is also the coordinating hospital of the regional FAST stroke network (Schlaganfallkonsortium Rhein-Neckar, *www.fast-schlaganfall.de*). FAST was established with the aim to improve cooperation around EVT provision, including emergency medical services, and to ensure EVT coverage also outside of the immediate catchment areas of EVT hospitals. The network covers parts of three federal states, and (at the time of data collection) included five EVT hospitals, eight IVT hospitals with SU, and eight cooperation hospitals focusing on post-acute care. Within the network, telestroke consultations were provided by the coordinating EVT hospital to seven IVT hospitals.

### Research design and study perspective

As described above, the aim of the study was to capture and make visible the perspective of patients with acute stroke along their pathway through the hospital(s) so as to portray what they would experience and perceive. However, given that many acute stroke patients are not conscious (enough) to be aware of everything that happens to or around them, this means that we were looking for information that patients could perceive in theory but usually not in practice. As a consequence, even though patient and relative interviews were conducted within the scope of the larger evaluation of which this study was part, these were not used for the current analysis. This was primarily due to considerable patient memory gaps, structural absence of relatives from important parts of the pathway and ambiguities in recollections that could not be attributed with reasonable certainty to specific locations or events. As a proxy, we therefore combined the two most adjacent perspectives, namely the perspective of staff members through staff interviews and the perspective of observers who followed the patients along their pathway.

### Stakeholder involvement

Even though we could not use the patients’ and relatives’ experiences as a primary source, we aimed to ensure the involvement of the stakeholder perspective by discussing the preliminary results of this study and suggestions for practice implementation with the Patient Council of the Department of Neurology on 3 March 2020. These insights are considered in the discussion section. For the patient and relative interviews (which are not considered in this study), members of a local stroke self-help group had provided advice on the research design and helped pilot the interview guides. This was of relevance for the results of the larger evaluation published elsewhere [49,50], and also considered in this study’s discussion.

### Data collection

Recruitment and data collection took place between March and June 2018. The core team responsible for data collection and analysis was not involved in patient care. It consisted of a social scientist experienced in qualitative health research (LB) and two Master students with health professional backgrounds (JH, CS). At the time of the research, JH was a nurse and CS a speech therapist, neither of whom was employed at the department under study, and both of them conducting research within the scope of their Master’s thesis for the program “Health services research and implementation science in health systems”. They had no prior relationship to the staff members they encountered during the observations nor to the interviewees. Throughout the phase of data collection, they were introduced to key contact persons primarily by their supervisors LB and CG who worked at the case site. Additionally, JH and CS were contacted and initiated contact with various staff members throughout the observation period, several of whom were approached for interview participation, as also described below. LB works at the department under study as scientific coordinator and researcher. CG is a senior physician who is primarily responsible for the hospital’s Stroke Unit. He is also head of the research group “health services research in neurology”.

Regular research team meetings took place at least once a week or more often when relevant questions arose, especially during the period of data collection and analysis. The team meetings were used to update each other on which data had been collected, how it had been recorded, how it had been perceived and interpreted by the researchers, whether questions or comments had been received from patients or colleagues (e.g. related to the interviews and observations or the research project more generally), and whether researchers thought that the methods of data collection (or later, data analysis) needed to be adjusted. The research team meetings were also the forum to discuss and decide whether and in which areas saturation had been achieved and no further data collection would be necessary.

Another important aspect discussed during team meetings was the recruitment of potential interviewees, especially when initial contact was made during observations. At times, this occurred when staff members approached researchers either to generally make their views known or to specifically express interest in the interviews after they had heard or read about them. Other times, the researchers identified potentially relevant interview partners during their observations, for example because their place of work or specific tasks where especially salient to the research purpose, or they shared relevant information or opinions during the observations that researchers wanted to collect more details on during an interview. In those cases, the potential interview candidates were discussed, compared to the purposive sampling strategy and the current status of recruitment, and a decision was taken on whether or not to ask them to participate.

### Observations

#### Before the observations

Staff members working at the relevant observation sites were informed in advance about the research project, the methods of data collection and how exactly the data collection would take place at their department or ward. Usually, this process started by contacting the senior physician of the relevant ward by email, providing an information leaflet on the project, followed by a meeting with the senior researcher (LB) during which the project and methods were explained and questions could be asked and answered. This was then followed by further announcements and information provision, usually by the senior physician(s) to their teams (including staff members of all professions), usually first by email followed by informal conversations. These are all information provision channels that are regularly used by the ward teams for all types of research-related and clinical information provision. In this context, it is common that different channels need to tapped, for example because many nurses tend to not regularly check their work email. Moreover, direct personal and phone conversations are often the preferred mode of communication among staff.

However, this process was not always uniform, primarily because of the different levels of prior information regarding the research methodology as well as the research project in general. When staff members were part of the project team around the implementation of the FAST stroke network, they tended to be more aware and informed about the research plan as well. During the brief overlap of when the observation phase ended and the interview phase started, it occasionally occurred that a physician had already been approached for or participated in an interview before granting access to “their” department or ward.

There was a direct feedback loop for the researchers to “check” whether the information provision had been effective and pervasive, as they noticed that their presence was expected when they arrived for the first observations. During the observations, when nothing of relevance to acute stroke care happened, staff members regularly approached the researchers to ask more detailed questions about the study background of the researchers, what type of information they were writing down, what would happen with the information, or to spontaneously provide the researchers with more background on what was happening around them. When the more junior researchers (JH, CS) were not sure whether they had been able to (adequately) answer all questions, this was discussed in the research team meetings, and sometimes additional information was provided by the senior researchers to staff members, or a conversation was offered if someone wished to ask more questions.

#### During the observations

Non-participant observations took place between March and May 2018. “Non-participant” meant that the researchers were present at the observation site, for example sitting in the physicians’ room and following physicians or nurses on their ward rounds or to patient rooms. However, they took great care not to influence or interrupt what was naturally occurring. This was not only relevant for the correct implementation of the intended methodology, but also a prerequisite for researchers’ presence at a working ED where health personnel were taking care of acutely ill patients.

Whenever possible, observations were conducted by two observers (JH, CS and/or LB). Deviations from this rule only occurred when several relevant events took place at the same time (e.g. two patients with suspected stroke arriving within minutes of each other) or when only one observer was available at short notice (e.g. when the researchers were notified of a stroke patient arriving or a specific procedure taking place within the next minutes). During all observations, both researchers took field notes to record information on the physical layout, people involved, activities observed, personal interactions, sequencing of events and (where possible) the emotions expressed. After transcribing both researchers’ field notes into chronological observation protocols describing what had happened when and where, the two protocols were consolidated into one.

As described above, during research team meetings the methods of data collection were constantly monitored, reflected and discussed to allow for possible adaptations. With regard to the observations, one of the adaptions was related to whether it was feasible and productive for the research aim to only conduct planned observations with pre-specified durations and locations. The primary concern was that certain events, such as the arrival of a patient with suspected stroke and telestroke consultations, had already been observed repeatedly, whereas others, such as an EVT procedure, had not happened yet during those time frames. After conducting five of these planned observations with durations of four to five hours at the ED, an additional *ad hoc* observation system was therefore adopted. In this system, the ED neurologist informed the researchers via telephone as soon as the arrival of a patient with a suspected stroke was announced. This approach was also used in another study conducting observations of acute stroke events at an emergency department [51].

### Interviews

The interviews were conducted with a semi-structured interview guide developed and piloted by the research team. The initial version of the interview guide was developed based on the domains specified in the COMIC model, the domains specified in the establishment plan for the FAST stroke network, and the different stages and departments specified in the standard operating procedures for acute stroke care provision at the case site. Throughout the data collection process, the questions were adapted to the different professional backgrounds of the interviewees and experiences gained during the interviews. A pilot interview was conducted with a physician who was asked to provide feedback on the questions and other potentially relevant aspects (e.g. duration, atmosphere, relevance). Since there were only minor suggestions for changes, we decided not to conduct more pilot interviews, but instead to focus on adaptations of the interview guide for each of the different professional groups, as mentioned above.

The interviews were conducted between May and June 2018. Initial contact between the research team and interviewees was established throughout the observation period. As with the observations, potential interview partners were contacted using a variety of communication channels, including emails (with the aforementioned information leaflet for the study) and personal communication, both face-to-face and over the phone. Interviewees were selected using a purposive sampling method and stepwise recruitment process, aimed at recruiting at least one interviewee from each profession involved in the multidisciplinary acute stroke treatment of the EVT hospital [52,53]. These steps were continuously discussed, monitored and adapted during research team meetings, as specified above. The response rate was 100%. All interviews were conducted in German by one researcher (JH).

Audio-recordings were transcribed verbatim. The accuracy of the transcriptions was ensured by using professional transcription agencies. Additionally, the researcher (JH) who had conducted the interviews was also responsible for the first coding step, thereby re-reading the transcripts and correcting minor mistakes or specific medical terminology misinterpreted by the transcribers.

### Data Analysis

Consolidated observation protocols, interview transcripts and field notes were integrated into the final data analysis. Interview transcripts were made available to participants upon request, but no requests for changes were communicated to the research team. All interviews were analysed using the qualitative data analysis software MaxQDA (2018, VERBI, Berlin, Germany). To ensure a homogeneous interpretation of the interviews, two authors (JH, LB) participated in the analysis, using an open coding process based on the interview guides, the COMIC Model [46] and the constant comparative method based in a grounded theory approach [47]. In regular consultations within the research team the coding process and scheme were discussed and adapted if necessary.

Within this study, we analysed the phase from admission at the EVT hospital until transfer or discharge from the EVT hospital. Findings were categorised chronologically along the care chain and according to the location of care provision and health professional group with direct patient interactions, as this would provide information on which parts of the pathway would be visible to patients. We defined this as face-to-face contact (including via video-link) between a staff member and the patient, even though depending on the patients’ status, they may or may not have been aware of this. Even though patients were interviewed as part of the larger study, this data was not considered in the current analysis due to gaps and ambiguities in their recollection of the precise pathways. Finally, the current analysis did not consider medical or other aspects of stroke care taking place “behind the scenes”, i.e. outside of the patient’s presence, such as multidisciplinary meetings or staff handovers between shifts. For example, neurologists consult neuroradiologists to interpret CT images or cardiologists who contribute to the evaluation of stroke aetiology. Though highly relevant to optimal stroke care provision, this is not directly visible to a patient along their pathway and was therefore not within the scope of this study.

## Results

We conducted twenty-seven observations, of which ten were planned, and seventeen *ad hoc*. The main location of twenty-two observations was the ED (including the angiography suite); three observations were (mainly) conducted at the SU; and two at the ICU. The observations lasted between 15 and 300 minutes. Fifteen semi-structured interviews were conducted with 16 health care professionals including neurologists, neuroradiologists, nurses, medical technical assistants and radiographers, social workers, occupational therapists, speech therapists and physical therapists. Two therapists preferred to be interviewed together. The interviews lasted between 37 and 97 minutes.

In the following we provide both visual and text-based descriptive overviews of the patient pathways during acute stroke treatment. They include a detailed focus on what happens (i.e. actions by different professional groups of staff members, patients, and potentially relatives), when it happens (especially in which chronological sequence and within which time frame), where it happens (e.g. at which type of hospital, department, and/or ward; but also whether in person or virtually), and who is involved (focussing on who interacts with whom).

### Patient pathways: what happens when, where and with whom?

**Figure 1:**
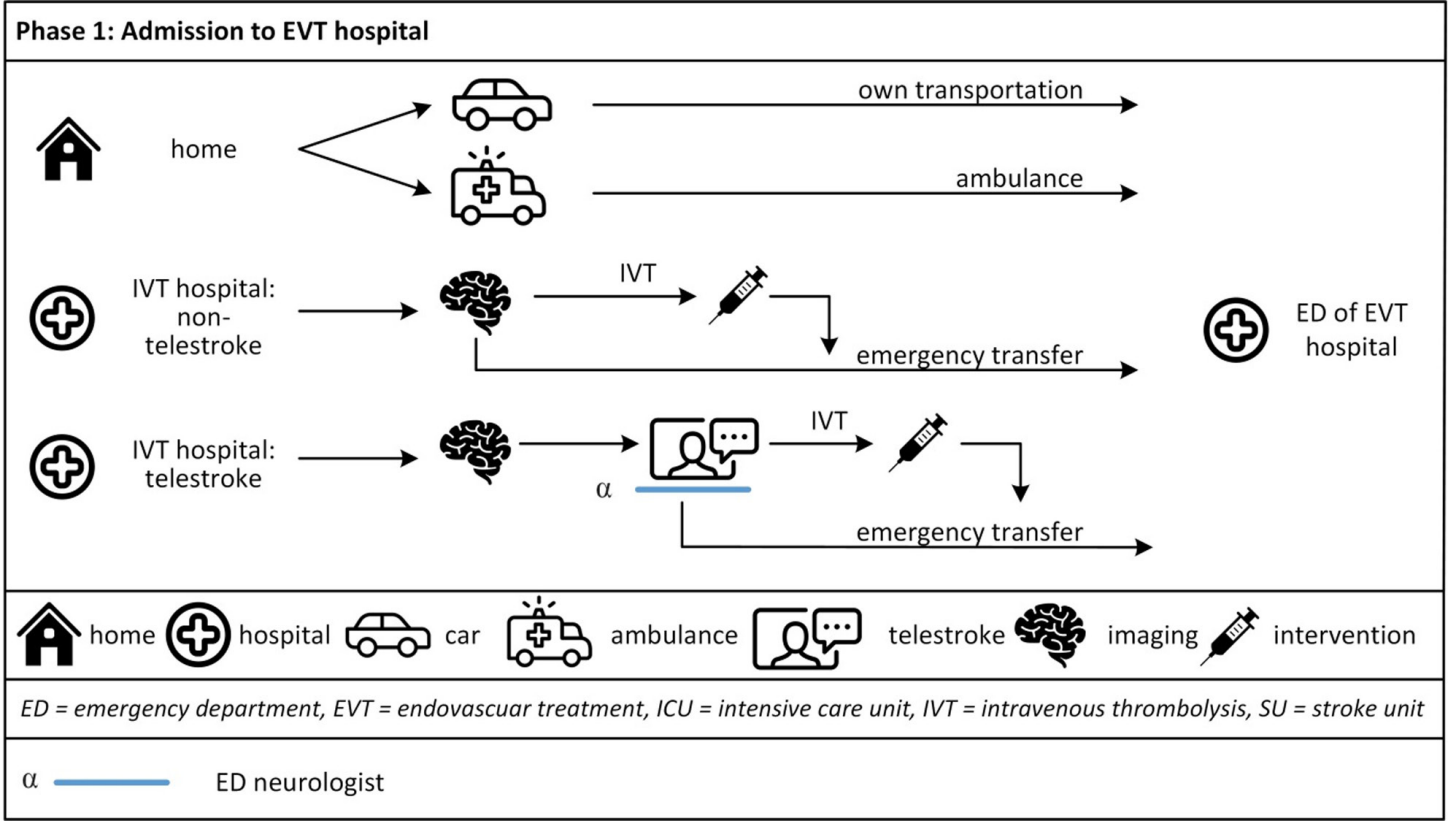
Pathways leading up to admission at EVT.

**Figure 2:**
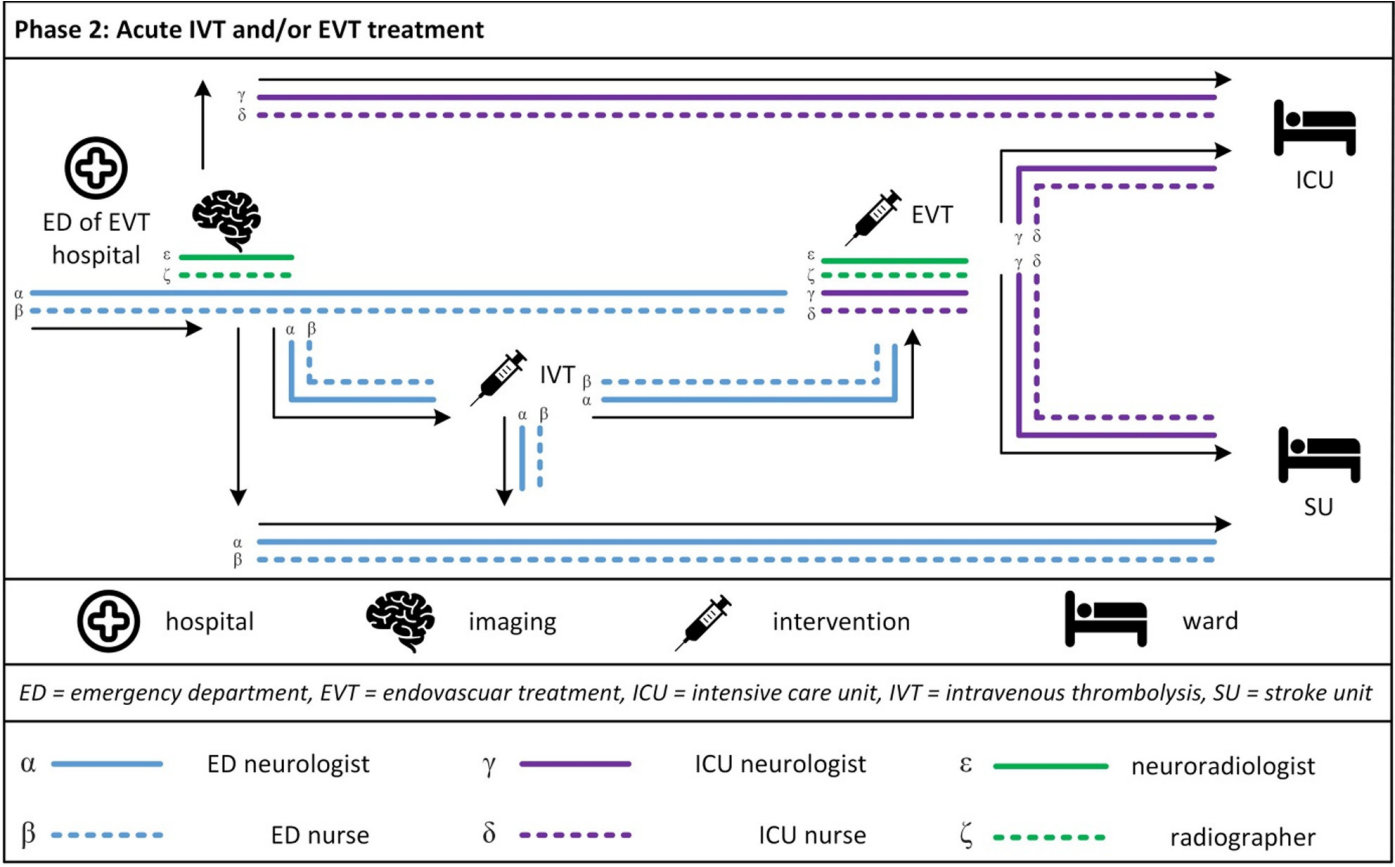
Acute IVT and/or EVT treatment.

**Figure 3:**
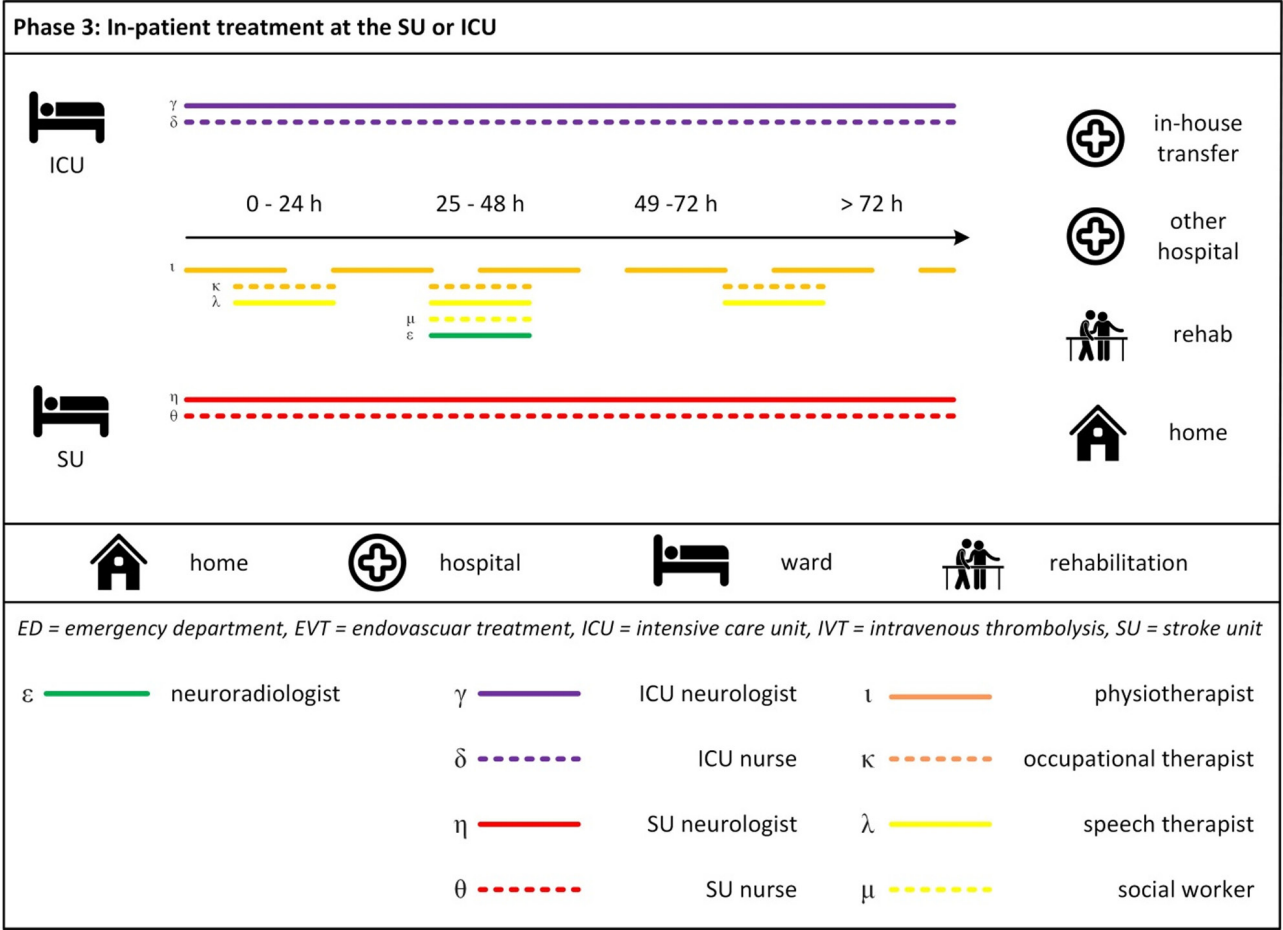
Pathways during in-patient treatment at the SU or ICU.

The patient pathways during in-hospital treatment were described along the phases (1) admission to EVT hospital, (2) acute IVT and/or EVT treatment, and (3) in-patient treatment at the SU or Intensive Care Unit (ICU). Along these pathways, direct staff-patient interactions were found to occur with members of five main health professional groups, namely the ED team, neuroradiology team, ICU team, SU team and the therapists including social workers, and at four locations, namely the emergency department, neuroradiology department, ICU and SU. Most direct staff-patient interactions were found to be focussed within one phase, with a smaller number extending to other phases, and with no professional group (of the same team) found to have direct patient interactions across the all phases. ***Figures 1, 2, 3*** provide a visualisation of the care provision per phase, location and health professional group.^1^

#### Phase 1: Admission to EVT hospital

***Figure 1*** shows the pathways leading up to admission at the EVT hospital including the contacts between EVT hospital staff and patients. The following admission options were observed or mentioned by the interviewees: patients going to the EVT hospital on their own, transportation by ambulance to the EVT hospital (primarily from the patient’s home or place of symptom onset, but also including care homes or other institutions), in-house transfer from a different department within the same hospital, and transfer from an IVT hospital to the EVT hospital. This latter option is called *drip and ship*, whereby the patient is evaluated for initial IVT treatment (“drip”) at the IVT hospital before being transferred (“shipped”) to the EVT hospital for further evaluation and treatment.

If the patient is initially admitted at an IVT hospital supported by the telestroke network, a telestroke consultation may be conducted. During the telestroke consultation, the first interaction between the patient and the ED neurologist of the EVT hospital takes place. As the ED neurologist speaks directly to the patient via a live video connection, both see and hear each other via camera and microphone. The neurologist assesses the patient with the assistance of the on-site physician of the IVT hospital, sometimes supported by a nurse. Depending on their health status, patients are encouraged to interact directly with the neurologist as they would in a face-to-face situation.

When patients have contra-indications for IVT or arrive outside or with an unclear time window (e.g. wake-up stroke) they can be transferred directly to the EVT hospital for EVT/IVT evaluation and/or provision.

#### Phase 2: Acute IVT and/or EVT treatment

***Figure 2*** depicts care provision around acute IVT and/or EVT treatment at the EVT hospital. When patients arrive at the ED of the EVT hospital, they are treated by the ED neurologist and one or two ED nurses. The ED neurologist informs the neuroradiologist as well as the ICU and SU staff as soon as possible about the arrival of a suspected stroke patient. Observed staff-patient interactions and communications at the ED were focused around the patient’s medical history, the neurological assessment, stroke onset time and the explanations of further treatment actions. The patient is continuously monitored by staff, and someone is always at the patient’s side. After history-taking is completed, medical imaging is conducted. The ED neurologist, who is primarily responsible for the patient at this stage, and an ED nurse accompany the patient to and throughout the procedure. At the time of entry into medical imaging, a neuroradiologist and a radiographer have their first face-to-face contact with the patient. In case of EVT indication, the patient is transferred by the ED staff to the angiography. Here the patient is handed over from the ED staff to the ICU staff, whose first interaction with the patient occurs at this point.

The ICU staff, which has been informed by the ED staff upon arrival of a patient with suspected stroke, is on alert but remains at the ICU until the indication for EVT is given. During one planned observation at the ICU, a suspected stroke patient was announced to arrive at the EVT hospital. It could be observed how nurses’ and neurologists’ focus and activities immediately and swiftly shifted to the upcoming EVT procedure. This sudden interruption of normal activities was also mentioned by interviewees. They described their regular and scheduled activities at the ward and how these would have to be abandoned or handed over at a moment’s notice when such an emergency call came in.

During EVT, patients are either intubated or in conscious sedation. The patient’s sedation and medical support are provided by one ICU neurologist and intermittently by one nurse of the ICU. The EVT intervention itself is conducted by one or two neuroradiologists with the assistance of one radiographer. Specifically, the radiographer is responsible for preparation of the sterile angiography desk, the groin and the position of the patient, while the ICU nurse and the neurologist monitor the patient, and the neuroradiologist focusses on catheter selection, access ways and the procedure itself. Interviewees mentioned that radiographers and neuroradiologist have low levels of verbal staff-patient interactions during medical imaging and EVT, describing that the main responsibility for patient care and interactions lies with ED and ICU staff. In contrast to these statements by the interviewees, conversations between the patient and the neuroradiologists in the angiography were observed several times. Of course, in these cases the patients’ vigilance influenced the intensity of staff-patient interactions during EVT. Compared to the verbal communication at the ED, staff members talked less often to the patient during EVT. Just as at the ED, the patient is continuously monitored throughout the EVT procedure, and an ICU neurologist is always at the patient’s side.

In most cases, patients are transferred to the SU, irrespective of whether an intervention (IVT or EVT) was conducted. If an EVT was performed, the transport is conducted by the ICU staff, if not, by the ED staff. When patients are intubated and/or require ICU monitoring, the patient is transferred to the ICU when the EVT procedure is over.

#### Phase 3: In-patient treatment at the SU or ICU

***Figure 3*** shows the pathways during in-patient treatment at the SU or ICU. At the SU or ICU, patients usually receive intensified neurological treatment by a multidisciplinary stroke team, typically for at least 72 hours. Neurologists and nurses work in a 24/7 shift system. Physiotherapists, occupational and speech therapist work from 8 am to 4 pm on the SU and the ICU and upon request on non-working days. Social workers, neuroradiologists and psychotherapists work on a consultation basis on the SU and the ICU on week days. The nurse is the first staff member of the SU in contact with the patient. Compared to other medical professionals, nurses spend the most time and intimate situations with the patient. As one physician pointed out, nurses are therefore often the first ones to notice changes in patients’ health status.

At least every six hours, the neurologists on the SU and ICU are in direct personal contact with the patient. During the daily rounds on the SU and the ICU, neurologist-patient interactions take place during which the neurologist conducts a specific neurological assessment and discusses treatment or transfer and discharge options with the patient. Patients were observed to ask questions and express treatment preferences. The neuroradiologist visits the patient one day after EVT to conduct a follow-up examination. Two of these follow-up examinations were observed, in which the neuroradiologist talked to the patient and answered questions about treatment.

Therapists are informed about the arrival of new stroke patients in the daily staff briefings or through informal conversations. The patient’s first contact with an occupational therapist, physiotherapist or speech therapist typically takes place in the afternoon of the day of arrival or the day after. These treatments are tailored to the individual needs of the patient and last between 20–45 minutes. Physiotherapists on the SU work with patients twice a day during the week. Interviewed therapists mentioned having more time for informal conversations than physicians or nurses and therefore being able to give more emotional support to the patient. This close contact between patients and therapists was also confirmed by other interviewees. Nurse-patient and therapist-patient interactions during diagnostic, therapeutic and care treatments were observed which often included informal and private conversations on a personal level between providers and patients. The first contact between the patient and the social worker usually takes place on the second day of the stay. The social workers’ activities include the arrangement of rehabilitation and/or home care after the patient’s discharge and other types of practical support. The frequency and intensity of the interactions varies according to patient-related factors. In general, the atmosphere at the SU and ICU in Phase 3 was observed as less hurried compared to the ED in Phase 2. Although SU and ICU staff are not continuously at the patient’s side, staff-patient interactions were observed to develop on a more personal level.

How long a patient stays at the ward depends on different factors including the patients’ health status, their home environment (e.g. whether they need to climb stairs or whether a caregiver is available) but also the ward’s occupation rates to allow for the arrival of new emergencies. When it is deemed safe and beneficial, patients may be transferred in-house to another ward, to an IVT hospital (usually the one that initially referred the patient to the EVT hospital), a rehabilitation facility, or discharged home. In case of rehabilitation, relatively younger patients are generally referred to neurological rehabilitation whereas relatively older patients will be referred to geriatric rehabilitation.

## Discussion

With this study we aimed to provide an overview of the patient pathways during acute stroke treatment at an EVT hospital of a regional stroke network from the patient perspective, including points of interactions with different health professional groups. In doing so, we aimed to centre the perspective of the patient alongside the medical and professional perspective, even though this had to be done by proxy (staff interviews and observations) due to relevant memory gaps and absences by patients and relatives, respectively. Our findings are reported in a way that allows for them to be used for information and education purposes aimed at patients, relatives and staff as well as for improvement efforts at the organisational level.

### Summary of main findings and comparison to other studies

We defined three phases of acute stroke care provision (admission at EVT hospital; acute IVT and/or EVT treatment, and in-patient treatment at the SU or ICU) during which five main health professional groups (ED team, neuroradiology team, ICU team, SU team and the therapists including social workers) at four locations (the ED, the neuroradiology department, the neurological ICU and the SU) have direct interactions with the patient. Before admission to the EVT hospital, interactions between staff members from the EVT hospital and patients take place as part of the telestroke consultations during which the EVT hospital’s ED neurologist meets the patient via a video- and audio-based connection. During the second phase, when IVT and/or EVT are provided to the patient, three teams (ED, neuroradiology and ICU team) with direct patient interactions intersect at the angiography suite until mechanical recanalisation treatment ends and the patient is transferred to the SU or ICU. In the third phase, the patients are treated on the SU or ICU and staff members interact with them according to a pre-defined schedule as well as based on individual needs.

Our results show that most direct staff-patient interactions are focussed within one phase. Only a smaller number of interactions span more than one phase: the ED neurologist has their main activity in Phase 2 *plus* a telestroke consultation in Phase 1; the neuroradiologist has their main activity in Phase 2 *plus* a follow-up consultation in Phase 3, and the ICU team is active in Phase 2 and 3 *if* the patient receives EVT and cannot breathe spontaneously afterwards. Finally, we found no professional (group) whose direct patient interactions cover more than two phases of the acute stroke pathway.

We are aware of two other qualitative studies reporting stroke patient pathways. Both studies were conducted prior to the implementation of EVT in clinical routine, which fundamentally changed organization of stroke treatment (as explained in ***Information box 1***). The first study concerns an analysis of an integrated stroke strategy in Alberta, Canada, spanning the pathway from community to hospital admission back to community integration [54]. In addition to the professional groups and locations reported here, theirs also included general practitioners, the community setting, and as transfer destinations, nursing homes, inpatient and outpatient rehabilitation facilities and stroke prevention clinics. Relevant differences in the pathways include the use of early supported discharge teams to support patients’ transition from hospital to community-based care and follow-up consultations at stroke prevention clinics. These built-in approaches to outreach from hospital into the community (or from acute to long-term care) could provide an interesting template for our case site, as could their involvement of stroke survivor representatives in the creation of the stroke strategy, which was not the case at our case site.

In their 2014 study, Davoody et al. describe collaborative interaction points between stroke patients, relatives and health professionals in post-discharge stroke care in Stockholm County, Sweden, spanning the pathway from Stroke Unit/Emergency Hospital to different types of rehabilitation modalities to primary and community care [55]. Additional stakeholders that differ from our findings include district nurses, patient organisations and municipal family supporters. Moreover, they differentiate patients able to visit health centres from those requiring home visits. This allowed them to identify different process flows by patient need and, therefore, to propose distinct recommendations for practice improvements. Finally, the Swedish study reported so-called “intersection points” between different care professionals providing collaborative care [55]. This seems to be a useful focus to also investigate those interactions occurring “in the background”, i.e. without the patient being present, which were not considered in our study.

### Relevance and implications for research and practice

By assessing what is usually framed as a purely medical intervention from an integrated care perspective, this study points towards potential areas for future research and practice improvements. In the following, we outline the relevance of our findings for patients and relatives, staff, hospitals, and researchers in acute stroke and/or integrated care.

#### Patients and relatives

As explained above, we had originally planned to base this study on patient and relative interviews as well as staff interviews and observations. However, our experience showed that many parts of the patient pathway are not visible to patients and relatives in real time. Nor were they able or had they tried to access this information after their discharge, i.e. at a time when they would have been more likely to understand and retain the information provided. In the interviews (conducted as part of the larger evaluation), several patients could not tell the interviewer whether they had undergone a telestroke consultation or whether they had been treated with IVT and/or EVT [49,50]. Moreover, several interviewees expressed to the interviewer that the interview (conducted approximately one month after stroke) was the first time they had talked about their experience since it had happened. As several interviews were conducted together with the patient and their relative, the conversations sometimes revealed that it was also the first time the patient heard their relative’s version of events, and vice versa.

In terms of direct practice improvements from the patient and relative perspective, the results of this study should therefore be translated to accessible patient information resources providing insights into what happened to the patient during their journey through hospital(s). This could be realised both to provide information on the *general* stroke pathways available to stroke patients, as well as serving as the basis for more *individualised* information provision to individual patients. For example, the building blocks provided here can be customised to generate a personalised depiction of a given patient’s journey through the hospital system. Specifically for our case site, we used this study’s findings as the basis for the creation of an information leaflet for patients and relatives about the common care pathways in acute stroke care, as the analysis showed an information gap regarding this issue. To this end, we translated our findings to German, worked with our Patient Council to discuss the content and find accessible ways to phrase complex information, and added pictures (e.g. of our SU, the medication used for IVT and catheters used for EVT). In a currently ongoing pilot phase, this leaflet is provided to all stroke patients at the SU of this study’s case site. For future versions, the pathways could be complemented with the “background” elements that were not the focus of the current study, such as handovers or consultations among colleagues (i.e. the “intersection” points in the Davoody et al. study [55]) which do not take place in the patient’s presence or may not be consciously experienced by them. This may contribute to an increased reassurance of patients and their relatives that they were provided with high quality and multidisciplinary care even if this was not directly visible to them.

#### Interaction between patients, relatives and staff

Given that we found most professionals or professional groups to have direct patient interactions *within* a given phase of care, the question arises how this may affect interactions with patients and relatives *across* phases of care. For example, most patient requests for information are likely to emerge at the SU where patients tend to regain (full) consciousness, but SU staff do not have direct patient interactions in the two previous phases. To this purpose, acute care may look at integrated care initiatives from the area of chronic care, in which the use of such strategies to support communication needs – for example, the use of care coordinators operating across institutions, shared medical records or designated family contact persons – may already be more common [56,57,58,59,60,61]. Similar questions may also be asked regarding communication across different hospitals and other institutions such as long-term care facilities. Future research, especially with a more extended focus on the networks (such as FAST) within which acute stroke care is increasingly being provided, could shed light on how communication and cooperation building has changed across institutions as stroke care provision has become more complex and how this affects the objective and experienced quality of care. Within FAST, for example, EVT patients are provided with a written information leaflet on why their transfer to and from the EVT hospital was necessary and explaining the partnerships within the network. However, its impact on the patient experience as not yet been evaluated. Future research should investigate whether or how the organisation of care may affect information flows from the perspective of staff, patients and relatives, and if so, the necessity and effectiveness of appropriate mitigation strategies.

#### Staff

Similarly to the previous point, our results have shown that most professional groups work within one phase of care which means that important parts of the pathway are not visible to all health professionals, either, depending on where they work. For example, therapists working at the SU may not have detailed knowledge about how a telestroke consultation is conducted or what exactly happens during EVT. As explained above, standard operating procedures (SOPs) are available to all staff, and again depending on where they work, must be known by them in detail. However, this is true to a lesser extent for SOPs outside their primary phases of care or outside of their profession. Moreover, as explained above, SOPs focus on medical aspects and are generally written from the provider perspective and without input from patients and relatives. For these reasons, the pathway descriptions generated in this study can be used to provide information and training to staff members regarding those parts of the stroke pathway that occur outside of their area of expertise and phase of care provision, but which may be relevant for their ability to provide high-quality stroke care across the care continuum, including both the perspective of their colleagues who work in other phases of care, as well as the patient perspective.

#### Hospitals

From the hospital perspective, our study provides relevant insights into the provision of care from a non-medical perspective. The above shows that even when acute stroke care is highly structured and explicitly geared towards achieving optimal treatment outcomes for patients, this does not automatically mean that it is well integrated and organised around the patient and their needs. Instead, if a hospital’s goal is to provide a service that is well integrated and organised around patient needs, the service must be explicitly designed, implemented, monitored and evaluated at such. This could also include structural mechanisms of how system “failures”, negative experiences or suggestions for improvement from service providers and services users can reach leadership. In this sense, being able to “see” current acute stroke provision along the route of a hypothetical patient going through it can help identifying non-medical areas for improvement that would remain invisible using only “traditional” medically focused process and health outcomes evaluations. Moreover, the structured process descriptions of the points of staff-patient-interactions may also be used for other improvement purposes, such as department- or institution-level digitalisation efforts. For example, the pathways could provide the basis for the creation of “digital twins” for more detailed and interactive depictions of the status quo as well as future improvement efforts.

#### Research in acute stroke and integrated care

As mentioned above, our research is one of the few studies with an integrated care perspective to investigate the acute phase of stroke care. When compared to studies focusing on (the transition to) longer term care, acute studies seem to be more centred on attainment of health outcomes or certain cost measures rather than patient or staff satisfaction [62,63,64,65]. Moreover, acute interventions tend to have a stronger focus on the organisational and provider side rather than on aspects such as person-centeredness, patient involvement or shared decision-making [62,63,64,65]. There are a few recent studies investigating these latter aspects in acute stroke care [66,67,68,69]. However, they were not specifically conceptualised or investigated as (components of) integrated care, and only two of them were conducted since the implementation of EVT into routine care [49,51]. What this suggests is a relevant mismatch between the astonishing pace at which the provision of acute stroke care has changed in practice vs. how this is being investigated. Especially when the provision of acute stroke care started to become increasingly network-based, this could have been understood as a call to action for investigations with a broader lens than only the medical. Instead, it seems that the areas of acute stroke research vs. integrated (chronic) care have remained relatively separate. This is also visible in core literature on integrated care, including those reported in recent reviews, whose primary focus does not tend to focus on acute stroke or acute care more generally [25,70,71,72,73,74,75]. In this sense, our paper provides a useful case study on how these two fields can complement each other to provide comprehensive scientific insights as well as avenues for practice improvements.

In conclusion, this study provides an overview of the status quo of how acute stroke pathways are currently organised and coordinated at a German hospital providing network-based stroke care. Thereby it also points out in which areas improvements or more insights are necessary. These potential improvements could support an organisation of acute stroke care that is not only well-coordinated from an organisational perspective but aims to work towards the definition of integrated care as developed by the patient organisation National Voices: *“I can plan my care with people who work together to understand me and my carer(s), allow me control, and bring together services to achieve the outcomes important to me”* [76].

### Strengths and limitations

Due to the single centre study design, several pathway sections such as emergency services, patient transfers or treatment in other institutions could not be considered in the current analysis. This also holds true for descriptions outside the main pathways, including possible night or weekend variations, or other health professional groups (such as cardiologists) providing relevant care that was however outside of the scope this specific analysis. Another limitation concerns our decision to not use the patient and relative interviews conducted as part of the larger study, mainly due to considerable patient memory gaps, structural absence of relatives from important parts of the pathway and ambiguities in recollections that could not be attributed with certainty to specific locations or events. The patient and relative interviews did hold valuable information on other aspects of stroke care, related more to experiences and opinions rather than exact descriptions of in-hospital processes, which will be analysed and reported separately to build on the descriptive results reported here. For the current analysis, we tried to remedy this shortcoming by reporting preliminary findings to our Patient Council who did not point out missing elements from their perspective. It should be noted again that our study did not focus on staff interactions or aspects of stroke treatment that did not involve direct patient interactions, such as medical handovers, staff meetings or consultations with colleagues. This means that no inferences should be made from our findings as to the degree of inter-professional cooperation, the continuity of medical information flow or the quality of medical care provided. Conducting a two-part methodological approach, including non-participant observations and semi-structured interviews, allowed us to triangulate findings and thereby obtain a more comprehensive overview of the non-medical aspects around acute stroke treatment. Our use of qualitative methods as well as an integrated care lens to study acute medicine is a relatively rare approach that allowed us to gain insights into the gaps in more traditional quantitative, medicine- and provider focused evaluations. As outlined above, our study’s explicit focus on non-medical, care-related and layperson-accessible aspects of acute stroke care means that findings can be translated relatively easily into patient information leaflets and staff education resources.

## Data Accessibility Statement

No data are available. As per the applicable ethics committee’s stipulations, we cannot provide access to observation protocols and interview transcripts. Reuse outside the research team is also not permitted as per the applicable ethics committee’s stipulations.

## Footnotes

Small variations in the illustrated stroke care pathways exist due to different telestroke contracts and varying structures of the hospitals in the stroke network as well as individual patient-related factors. The “ship the doc” concept, in which a neuroradiologist is brought to another hospital to provide EVT, was only mentioned by one neurologist and one radiographer and was never observed, and is therefore not shown. Specific pathway adjustments at night or weekends are not shown either.

## Picture attributions (Figures 1, 2, 3)

House by Guilherme Furtado from the Noun Project

First Aid by Viktor Ostrovsky from the Noun Project

Brain by Cédric Villain from the Noun Project

Syringe by David from the Noun Project

News by Gregor Cresnar from the Noun Project

Bed by Tara Nadhifa Salsabila from the Noun Project

Physiotherapy by Gan Khoon Lay from the Noun Project
